# Whole-cell conversion of l-glutamic acid into gamma-aminobutyric acid by metabolically engineered *Escherichia coli*

**DOI:** 10.1186/s40064-016-2217-2

**Published:** 2016-05-11

**Authors:** Chongrong Ke, Xinwei Yang, Huanxin Rao, Wenchao Zeng, Meirong Hu, Yong Tao, Jianzhong Huang

**Affiliations:** National Engineering Research Center of Industrial Microbiology and Fermentation Technology, College of Life Sciences, Fujian Normal University, Fuzhou, 350108 Fujian China; CAS Key Laboratory of Microbial Physiological and Metabolic Engineering, Institute of Microbiology, Chinese Academy of Sciences, No. 1 West Beichen Road, Chaoyang District, Beijing, 100101 China

**Keywords:** Gamma-aminobutyric acid, Glutamate decarboxylase, *Escherichia coli*, Whole-cell biocatalyst, Bioconversion

## Abstract

A simple and high efficient way for the synthesis of gamma-aminobutyric acid (GABA) was developed by using engineered *Escherichia coli* as whole-cell biocatalyst from l-glutamic acid (l-Glu). Codon optimization of *Lactococcus lactis* GadB showed the best performance on GABA production when middle copy-number plasmid was used as expression vector in *E. coli* BW25113. The highest production of GABA reached 308.96 g L^−1^ with 99.9 mol% conversion within 12 h, when *E. coli* Δ*gabAB* (pRB-lgadB) concentrated to an OD_600_ of 15 in 3 M l-Glu at 45 °C. Furthermore, the strain could be reused at least three cycles in 2 M crude l-Glu with an average productivity of 40.94 g L^−1^ h^−1^. The total GABA yield reached 614.15 g L^−1^ with a molar yield over 99 %, which represented the highest GABA production ever reported. The whole-cell bioconversion system allowed us to achieve a promising cost-effective resource for GABA in industrial application.

**Electronic supplementary material:**

The online version of this article (doi:10.1186/s40064-016-2217-2) contains supplementary material, which is available to authorized users.

## Background

Gamma-aminobutyric acid (GABA) is a four-carbon non-protein amino acid that is ubiquitous in bacteria, plants and vertebrates (Diana et al. 2014). GABA has been used extensively in functional foods and pharmaceuticals, because it can act as an efficient neurotransmitter in vertebrates (Wong et al. 2003). In addition, GABA can converted into 2-pyrrolidone, an intermediate in the synthesis of nylon 4 and agrochemicals, which broaden its industrial applications (Park et al. 2013; Yamano et al. 2013). Nowadays, the majority of the GABA was chemically synthesized from 4-chlorobutyronitrile, 2-pyrrolidone or 4-butyrolactone, however, chemical synthesis often resulted in environmentally unfriendly and cost-ineffective purification (Additional file 1: Table S1). Therefore, a strategy for economical production of biologically produced GABA for an industrial scale is eagerly demanded.

GABA is a natural metabolic intermediate in organisms, which can be synthesized via decarboxylation of glutamate by glutamate decarboxylase (GAD; EC 4.1.1.15) (Dhakal et al. 2012). There are many reports on the production of GABA by direct fermentation using natural or recombinant microorganisms (Pham et al. 2015; Shi et al. 2013). Among them, lactic acid bacteria (LAB) who contain inherent GABA synthesis pathway, produced high level of GABA in MSG-containing medium (Kook and Cho 2013; Li et al. 2010). However, an accompanying separation processes needed to be developed, and these processes were too complex to increase the cost of GABA purification (Kang et al. 2013). Recently, GABA could efficiently converted from glutamate (MSG) and l-glutamic acid (l-Glu) using purified GAD or microorganisms expressing GAD (Kang et al. 2013; Lammens et al. 2009). Together with high efficiency of MSG/l-Glu fermentation, bioconversion of MSG/l-Glu into GABA was more economically than the fermentative methods (Hermann 2003).

Glutamate decarboxylase, a pyridoxal 5′-phosphate (PLP)-dependent enzyme, is a key factor for the bioconversion. Due to its role in bacterial glutamate-based acid resistance system, most natural GADs exhibited their highest decarboxylase activity only under the acidic conditions (De Biase and Pennacchietti 2012; Kanjee and Houry 2013). This characteristic of pH-response was disadvantageous to GAD used in producing GABA, because the pH increase as the reaction proceeded would inactivate GAD and limited the conversion (Gut et al. 2006). Since the crystal structures of *E. coli* GADs revealed the structural basis for its optimal activity at acidic pH, several mutants with high activity toward more alkaline pH values have been constructed to improve GABA production (Choi et al. 2015; Shi et al. 2014; Thu Ho et al. 2013). On the other hand, hydrochloric acid, sodium acetate buffer and acidic cation-exchange resins were used to maintain the acidic condition during reaction from MSG into GABA, and the conversion efficiency were remarkably improved (Dinh et al. 2014; Park et al. 2013; Plokhov et al. 2000). However, these methods were also unsatisfactory for the following separation and purification of GABA due to the introduction of the high amount of salts or resins. In recent years, l-Glu was widely applied as substrate for keeping the acidic pH in non-buffered reaction with purified or immobilized GAD (Kang et al. 2013; Lammens et al. 2009; Yamano et al. 2013). Despite of the high conversion yield and the simple downstream separation, it was not suitable for industrial scale because of the tedious preparation of purified GAD and the requirement of expensive cofactor PLP.

In comparison to the purified enzymes method, whole-cell bioconversion is an attractive way due to its great efficiency, relatively easy preparation and low cost, which is of particular interest for large-scale applications (Schuurmann et al. 2014). In that case, *E. coli* was the most common whole-cell baiocatlyst (Tam et al. 2012; Vo et al. 2013; Yamano et al. 2013). For example, Plokhov et al. used recombinant *E. coli* strain as whole-cell biocatalyst, 138 g GABA was achieved from 200 g l-Glu at a conversion yield of 98.5 % (Plokhov et al. 2000); Naoko et al. used *E. coli* NBRC 3806 as the whole-cell biocatalyst, 303.7 g GABA was produced from 560 g l-Glu via repeating 14 times (Yamano et al. 2013). Except for *E. coli*, *Bacillus subtilis* and *Lactobacillus brevis* resting cell were also used as the whole-cell biocatalyst, however, the production was not so attractive for industrial scale (Zhang et al. 2012, 2014). Moreover, the presence of the cell envelopes was able to stabilize the intracellular glutamate decarboxylase and made it less material adsorption.

In this study, we constructed a recombinant *E. coli* to produce GABA by overexpressing *L. lactisgadB*. Then, an efficient whole-cell biocatalytic process for GABA production from l-Glu was developed by optimizing reaction temperatures, biocatalyst and substrate concentrations. The competing pathway was removed to reduce the degradation of GABA. Finally, crude l-Glu provided by Wuyi Gourmet Powder Factory was used for converting into GABA to investigate the possibility of connecting the whole-cell biocatalytic process with the actual MSG production process.

## Methods

### Bacterial strains and plasmids

The plasmids and bacterial strains used in this study were listed in Table 1. *E. coli* BW25113 and its derivative strains were used for GADs expression and GABA production, while *E. coli* DH5α was used for gene cloning and plasmid maintenance. Plasmid pCP20 was used to remove the *kan* gene, and the P1 phage was used to delete the *gadB* gene in *E. coli* Δ*gadA* (Baba et al. 2006; Thomason et al. 2007).

**Table 1:** The strains and plasmids used in this study

Strains or plasmids	Descriptions	References
*E. coli* strains		
DH5α	*F* ^−^ *, endA1, glnV44, thi*-*1, recA1, relA1, gyrA96, deoR, nupGΦ80dlacZ*Δ*M15,* Δ*(lacZYA*-*argF)U169, hsdR17(rK* ^−^ *mK* ^+^ *), λ* ^−^	Lab stock
BW25113	*F* ^−^, Δ*(araBAD)567,* Δ*lacZ4787(::rrnB*_*3*_ *), λ* ^−^ *, rp h*-*1,* Δ*(rhaBAD)568, hsdR514*	Baba et al. (2006)
Δ*gadA*	JW3485, *gadA* null mutant of BW25113	Baba et al. (2006)
Δ*gadB*	JW1488, *gadB* null mutant of BW25113	Baba et al. (2006)
Δ*gadT*	JW2637, *gabT* null mutant of BW25113	Baba et al. (2006)
Δ*gadAB*	*gadA and gadB* null mutant of BW25113	This study
Plasmids		
pCP20	Flp^+^, *λ*cI857^+^, *λ* PR Rep(pSC101 ori)^ts^, Ap^r^, Cm^r^	Datsenko and Wanner (2000)
pYB1S	p15A ori, arabinose-inducible araBAD promoter, Str^r^	Lab stock
pRB1S	RSF1020 ori, arabinose-inducible araBAD promoter, Str^r^	Lab stock
pAB1S	colA ori, arabinose-inducible araBAD promoter, Str^r^	Lab stock
pDB1S	cloDF13 ori, arabinose-inducible araBAD promoter, Str^r^	Lab stock
pSB1S	pSC101 ori, arabinose-inducible araBAD promoter, Str^r^	Lab stock
pUB1S	colE1 ori, arabinose-inducible araBAD promoter, Str^r^	Lab stock
pYB-bgadB	pRB1S with *gadB* from *L. brevis* BH2	This study
pYB-pgadB	pRB1S with *gadB* from *L. plantarum* ATCC 14917	This study
pYB-lgadB	pRB1S with *gadB* from *L. lactis* IL1403	This study
pAB-lgadB	pAB1S with *gadB* from *L. lactis* IL1403	This study
pDB-lgadB	pDB1S with *gadB* from *L. lactis* IL1403	This study
pSB-lgadB	pSB1S with *gadB* from *L. lactis* IL1403	This study
pUB-lgadB	pUB1S with *gadB* from *L. lactis* IL1403	This study
pRB-lgadB	pYB1S with *gadB* from *L. lactis* IL1403	This study

### Construction of plasmids

Standard methods were used for PCR, ligation, plasmid construction, extraction of plasmid DNA and genomic DNA and transformation (Green and Sambrook 2012). DNA polymerases, restriction endonucleases, T4 DNA ligase, and vector were purchased from NEB (New England BioLabs, China).

The *gadB* genes were synthesized according to the sequences from genomic DNA of three different strains, including *L. brevis*, *L. lactis* and *L. plantarum* with codon optimization (GenBank accession AIC75915; AAK05388; EFK28268). Nucleotide sequences of three codon-optimized *gadB* genes were submitted to GenBank under the accession number KT966875, KT966877 and KT966876. *gadB* genes, with the restriction sites *Xho*I upstream and *Spe*I downstream, were digested by *Xho*I and *Spe*I, and then ligated into the plasmids.

### Gene disruption

The *gadA* and *gadB* double mutant was disrupted by P1 transduction (Thomason et al. 2007). Briefly, the phage P1 was grown on the donor strain Δ*gadB* containing the transferable elements, and the resulting phage lysate was used to infect the recipient *E. coli* Δ*gadA* strain. The *kan* gene was eliminated using the plasmid pCP20, which encodes the FLP recombinase. The mutant strain was confirmed by PCR amplification with primers (forward, 5′-TTAAACACGAGTCCTTTGC-3′ and reverse, 5′- AGCAGGAAGAAGACTAATGA-3′) and sequencing.

### *E. coli* cultivation in shake flasks

*Escherichia coli* strains were grown in LB medium (10 g L^−1^ tryptone, 5 g L^−1^ NaCl, and 5 g L^−1^ yeast extract) containing 50 mg L^−1^ streptomycin at 30 °C with shaking at 200 rpm. Then, 1 % pre-culture was transferred into 50 mL of ZYM medium (Studier 2005) with 50 mg L^−1^ streptomycin in 250 mL shake flask for GADs expression. Cells were cultivated at 30 °C for 16 h with shaking at 200 rpm.

### Fed-batch cultivation

For seed cultures, the *E. coli* was inoculated into 350 mL of LB medium containing 50 mg L^−1^ streptomycin in a 1 L flask at 30 °C for 8 h with shaking at 200 rpm. Then, seed cultures was transferred into 35 L of fresh basal medium (5 g L^−1^ yeast extract, 9 g L^−1^ KH_2_PO_4_, 4 g L^−1^ (NH_4_) _2_HPO_4_ and 0.6 g L^−1^ MgSO_4_) containing 20 g L^−1^ glucose and 50 mg L^−1^ streptomycin in a 50 L jar bioreactor. In the fed-batch cultivation, glucose concentration was maintained at 0.5 g L^−1^ in the broth to avoiding the glucose limitation. During the cultivation, temperature was maintained at 30 °C; pH was maintained at pH 6.7 by adding ammonia water; dissolved oxygen was maintained at 20 % (v/v) by automatically increasing the agitation speed up to 600 rpm with 1.0 vvm air flow rate.

### Bioconversion conditions

After induction, the cells were collected by centrifugation at 8000×*g* for 10 min and then resuspended in 0.1 M sodium acetate buffer (pH 4.6) with MSG or deionized water (DW) with l-Glu at appropriate concentration. The reaction mixtures were adjusted to different temperatures, cell densities and substrate concentrations to improve the GABA production. After the process was optimized, the cells were incubated at 45 °C with the addition of 3 M Glu to investigate the production of GABA. The same batch of cells were used three runs in 2 M crude l-Glu solution for each time to produce GABA. For each round of the cycling reaction, cells were harvested and adjusted to OD_600_ of 15, then mixed with l-Glu directly.

### Analytical methods

The cell density was estimated by measuring the optical density at 600 nm (OD_600_). One unit of OD_600_ corresponds to a wet cell weight of 0.83 ± 0.01 g L^−1^. Expression of recombinant GADs was analyzed by 12 % sodium dodecyl sulfate polyacrylamide gel electrophoresis (SDS-PAGE).

The concentrations of GABA and l-Glu were measured by HPLC with the phenylisothiocyanate derivation method, equipped with Hypersil GOLD C18 analysis column (250 mm × 4.6 mm, 5 μm, Thermo). The derivatization reagent consisting of phenylisothiocyanate/triethylamine/acetonitrile (0.02:0.18:2.3) solution (2.5 mL) was added to 2.5 mL of the supernatant. The mixture was incubated at 40 °C for 60 min. Then, 5 mL hexane was added to stop the reaction, shaken and aside for 10 min. The reacted solutions was analyzed at 40 °C using a linear gradient of two mobile phases (eluent A: acetonitrile; eluent B: 50 mM sodium acetate, linear gradient of 0–70 % eluent B in 15 min) at a flow rate of 0.8 mL min^−1^ and monitored at 254 nm (Takeda et al. 2012). The standard curves for GABA and l-Glu (Sigma, Missouri, USA) were determined using the same procedure.

## Results and discussion

### Construction of recombinant *E. coli* for the production of GABA

Initially, MSG was used as the substrate for whole-cell bioconversion of wild-type *E. coli*. However, only 7.86 g L^−1^ GABA was produced within 6 h, indicating the wild-type *E. coli* was not suitable as whole-cell biocatalysts for conversion into GABA. Thus, we constructed recombinant *E. coli* strains by overexpressing codon-optimized *L. lactis* GadB, *L. plantarum* GadB and *L. brevis* GadB from middle copy-number plasmid (pYB1S) under the regulation of P_araBAD_ promoter. The SDS-PAGE result showed the three *gadB* genes were successfully expressed, and the GadB proteins were produced with high solubility (Additional file 1: Fig. S1).

**Fig. 1:**
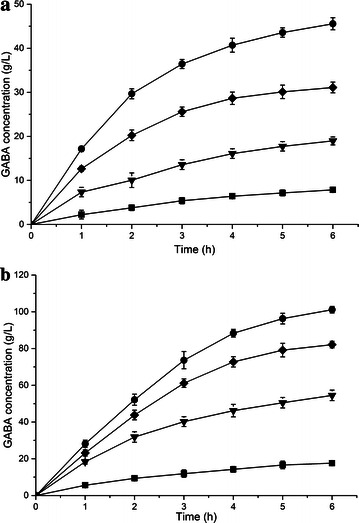
Time profiles of GABA produced in **a** MSG buffer and **b** l-Glu solution by *E. coli* BW25113 expressing different GadB. Conversions were performed at 37 °C and 25 g wet cell per liter. Data are presented as the mean ± SD values from three independent experiments. *Square*, *down triangle*, *diamond* and *circle* symbolize the *E. coli* BW25113 and recombinant *E. coli* BW25113 harboring pYB-pgadB, pYB-bgadB and pYB-lgadB, respectively

The effect of three candidate strains with different GadB genes on production of GABA was examined. We resuspended the strains with an OD_600_ of 30 in 0.1 M sodium acetate buffer (pH 4.6) containing 1 M MSG for bioconversion at 37 °C. As a result, recombinant *E. coli* BW25113 expresses *L. lactis* GadB was the most active whole-cell biocatalyst for GABA production, which resulted in the synthesis of 45.58 g L^−1^ GABA with a 44.2 mol% conversion within 6 h (Fig. 1a). The other two recombinant *E. coli* harboring *L. brevis* GadB and *L. plantarum* GadB produced lower GABA concentration of 31.12 and 18.97 g L^−1^, respectively.

### Glutamic acid is a good buffer for bioconversion

High concentration of MSG and GABA that contained ionizable alpha-amino groups made the bioconversion reaction maintain at near-neutral pH environment, which was beyond the active range of wild-type GAD. Instead of using buffer solution with the MSG in the reaction, we applied pure l-Glu to achieve acidic pH at the start of the bioconversion. Three recombinant *E. coli* strains produced GABA in water with adding 1 M l-Glu at one time. At the beginning, l-Glu was partially dissolved in water, then dissolved better as the conversion proceeded. Finally, l-Glu was dissolved completely in the system, which meant almost all of the l-Glu was converted into GABA. As we expected, the three strains with l-Glu as the substrate showed higher efficiency and higher molar yield of GABA production than using MSG (Fig. 1b). The highest production of GABA was achieved at 101.16 g L^−1^ (0.98 M) using recombinant *E. coli* expressing *L. lactis* GadB, with a 220 % improvement compared to that obtained in 1 M MSG buffer (pH 4.6) within 6 h. Based on these results, recombinant *E. coli* expressing *L. lactis* GadB was selected for further investigations on the production of GABA using l-Glu as the substrate.

### Synthesis of GABA by recombinant *E. coli* harboring different plasmids

**Fig. 2:**
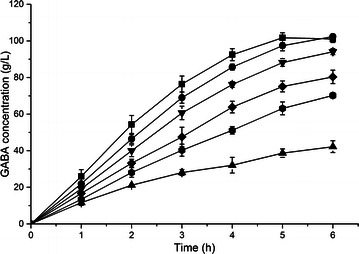
Production of GABA by recombinant *E. coli* harboring different plasmids. *Square*, *circle*, *down triangle*, *diamond*, *hexagon* and *up triangle* symbolize the recombinant *E. coli* harboring pRB-lgadB, pYB-lgadB, pDB-lgadB, pUB-lgadB, pAB-lgadB and pSB-lgadB, respectively

To provide appropriate expression level of *L. lactis gadB* in *E. coli* for GABA production, six different plasmids from a relatively low copy-number (~5) to high copy-number (~300) were applied. Recombinant *E. coli* BW25113 strains harboring pSB-lgadB, pAB-lgadB, pYB-lgadB, pRB-lgadB, pDB-lgadB and pUB-lgadB were investigated for the conversion of l-Glu into GABA (Fig. 2). To our surprise, the strain with the highest copy-number plasmid (*E. coli* BW25113 harboring pUB-lgadB) was only produced 80.33 g L^−1^ GABA within 6 h. The highest GABA production (102.19 g L^−1^) was achieved by recombinant *E. coli* harboring middle copy-number plasmid (pRB-lgadB) within 5 h with a space–time productivity of 20.44 g L^−1^ h^−1^. The result indicated a moderate rather strong expression of GAD was efficient for GABA production, and the soluble expression levels of GAD were not correlated with the production and the productivity of GABA (Additional file 1: Fig. S2). Moreover, the low copy-number indeed had the negative effect on GABA production, for example, recombinant *E. coli* harboring pSB-lgadB (the lowest copy-number) produced only 42.21 g L^−1^ GABA within 6 h.

### Optimization of bioconversion system for GABA synthesis

**Fig. 3:**
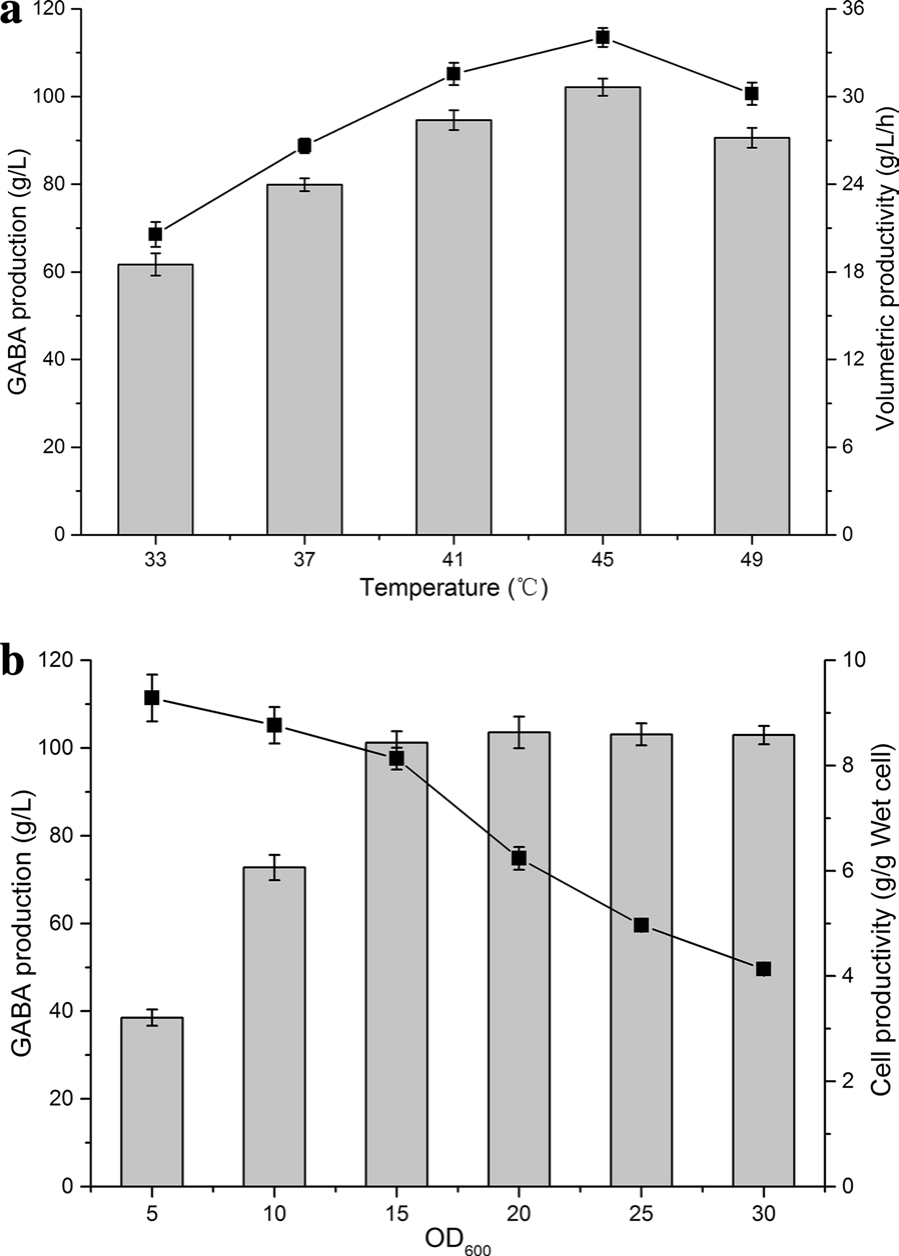
Effects of reaction condition on GABA production. Conversion reactions were performed at different **a** temperature and **b** cell concentrations with shaking at 200 rpm for 3 h. Data are presented as the mean ± SD values from three independent experiments. The *bars* represent GABA concentrations and the *square symbols* represent **a** volumetric productivity, **b** cell productivity

As previously reported, the temperature of the reaction was a core factor for glutamate decarboxylase activity (Lammens et al. 2009; Plokhov et al. 2000; Zhang et al. 2014). Thus, the conversion efficiency of *E. coli* BW25113 harboring pRB-lgadB was examined at five different temperature conditions (33, 37, 41, 45 and 50 °C) (Fig. 3a). The highest volumetric productivity of GABA (34.04 g L^−1^ h^−1^) was obtained at 45 °C, which was 1.67 times higher than previous study at 37 °C (20.44 g L^−1^ h^−1^). Consequently, 102.13 g L^−1^ GABA with a molar yield of 99.1 % was produced within 3 h at 45 °C, and this result consisted with the optimum temperature of purified GAD (EC 4.1.1.15) in *L. lactis* (Nomura et al. 1999).

To improve the total production of GABA during whole-cell bioconversion, the cell productivity was also investigated (Fig. 3b). Comparing with the strain concentrated to OD_600_ of 30 in previous study, the cell productivity of GABA was significantly improved as the cell amount decreasing. The cell productivity increased to a peak (9.28 g/g wet cell) using the strain with OD_600_ of 5, however, the GABA production only reached 38.53 g L^−1^ with 37.4 mol% conversion. Despite of a little lower cell productivity (8.13 g/g wet cell), the GABA production was achieved at 101.26 g L^−1^ with 98.2 mol% conversion using the strain with OD_600_ of 15. Furthermore,GABA yield only increased a little (1.71–2.33 g L^−1^) when the strain concentrated to OD_600_ over 15, while the cell productivity fell a lot (6.2–4.1 g/g wet cell). Therefore, the strain with an OD_600_ of 15 was more applicable for the whole cell bioconversion.

### Effect of substrate concentration on GABA production

**Fig. 4:**
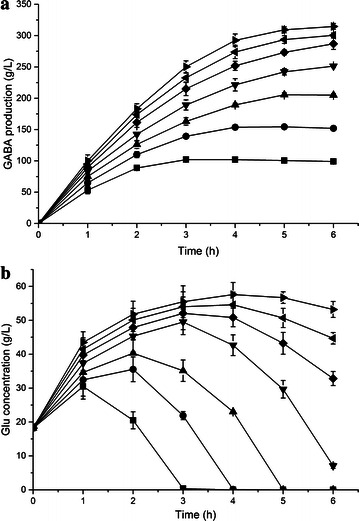
Time profiles of **a** GABA and **b** residual l-Glu concentration during conversion in different l-Glu concentrations. Conversions were performed at 45 °C and 12.5 g wet cell per liter (OD_600_ of 15). Data are presented as the mean ± SD values from three independent experiments. *Square*, *circle*, *up triangle*, *down triangle*, *diamond*, *left triangle* and *right triangle* represent the concentrations of l-Glu: 1, 1.5, 2, 2.5, 3, 3.5 and 4 M, respectively

**Fig. 5:**
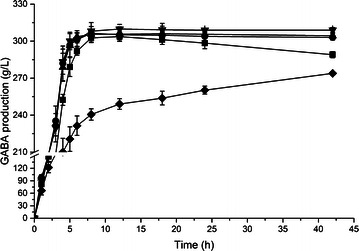
Time profiles of GABA concentration were obtained using various strains. *Diamond*, *square*, *up triangle*, *circle* and *down triangle* represent the recombinant *E. coli* Δ*gabT*, *E. coli* BW25113, *E. coli* Δ*gadB*, *E. coli* Δ*gadA* and *E. coli* Δ*gadAB* harboring pRB-lgadB, respectively

A series of l-Glu concentrations was used for whole-cell bioconversion to determine the influence of initial l-Glu concentration on GABA production. Time profiles of GABA production showed that the GABA yield increased with higher initial l-Glu concentration at the cost of conversion time (Fig. 4a). The most efficient system was controlling the initial concentrations of l-Glu less than 2.5 M, in that case, almost all of l-Glu was consumed within 6 h with producing an equal molar amount of GABA (Fig. 4b). Interestingly, the presence of GABA could promote the solubility of l-Glu, therefore, the concentration of l-Glu increased in the early stages (Additional file 1: Fig. S3). When the initial l-Glu concentration increased to 3 M, the conversion time prolonged to 12 h for consuming almost all the l-Glu, and the production reached 303.75 g L^−1^ with 98.2 mol% conversion (Fig. 5). Further increase in the initial concentration, l-Glu could not be completely converted to GABA even though longer conversion times (up to 24 h) was spent (data not shown). As a result, the highest volumetric productivity of GABA (41.12 g L^−1^ h^−1^) was achieved using 2 M l-Glu as the substrate, which resulted in the production of 205.61 g L^−1^ GABA with a conversion of 99.7 mol%. For the record, this is the highest volumetric productivity level of GABA produced by whole-cell bioconversion from l-Glu, even compared with conversion using the purified or immobilized GAD (35 g L^−1^ h^−1^) (Kang et al. 2013; Lammens et al. 2009).

### Effect of blocking competitive pathways in GABA production

It is worth noting that the GABA concentration strikingly degraded from 303.75 g L^−1^ obtained from 3 M Glu within 12 h to 289.07 g L^−1^ during 30 h (Fig. 5). GABA aminotransferase (GabT), which directed GABA to the TCA cycle, was considered to be the main cause of GABA degradation (Tam et al. 2012). Therefore, *E. coli* Δ*gabT* harboring pRB-lgadB was constructed as whole-cell biocatalyst to investigate GABA production. Recombinant *E. coli* Δ*gabT* indeed impeded the degradation of GABA, but it produced 273.96 g L^−1^ GABA by consuming 89 % of 3 M l-Glu within 42 h, which was only 25.9 % of the volumetric productivity obtained from the best strain (25.44 g L^−1^ h^−1^). The result was similar with previously reported, because *E. coli* Δ*gabT* caused a metabolic burdens, which lead to lower cellular metabolic activity (Tam et al. 2012).

Interestingly, when the chromosomal *gadA*, *gadB* or both were knocked out, the degradation of GABA was also prevented without influence the conversion yield. After 42 h, over 303 g L^−1^ GABA remained in the reaction solution with less than 1 % degradation when using the mutant *E. coli* Δ*gadA* or *E. coli* Δ*gadB* as host strain. Meanwhile, *E. coli* Δ*gadAB* harboring pRB-lgadB achieved the highest GABA concentration of 308.96 g L^−1^, and had only 0.25 % degradation of GABA for lasting 30 h. Based on these results, knocking out the chromosomal *gadA* and *gadB* gene in *E. coli* might be an efficient metabolic engineering strategy to prevent the degradation of GABA.

### Whole-cell biocatalysis of GABA from crude Glu

**Fig. 6:**
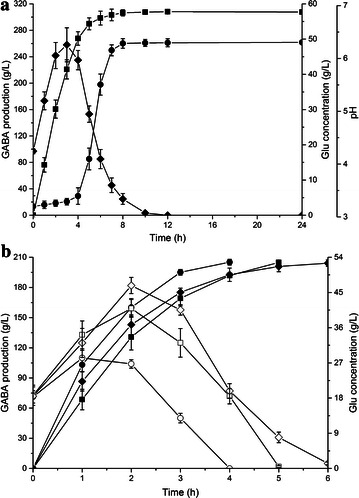
Time profiles of GABA synthesis from **a** 3 M and **b** 2 M crude l-Glu solution. **a** Time course where *diamond*, *square* and *circle* represent l-Glu concentration, GABA production and pH, respectively. **b** Repeated use of cells where *square*, *circle* and *diamond* represent cycle 1, cycle 2 and cycle 3, respectively; *filled shapes* GABA concentrations; *open shapes* residual l-Glu concentrations

Fed-batch cultivations was carried out to obtain a higher cell concentration (OD_600_ of 60) of *E. coli ΔgabAB* harboring pRB-lgadB. The bioconversion was performed in 200 L reactor by 15 OD cells suspended in 160 L deionized water with initial addition of 88.5 kg crude l-Glu at one time. Crude l-Glu, prepared from actual glutamate production process, was provided by Wuyi Gourmet Powder Factory. The pH of reaction mixture increased from 3.21 to 6.31 during 12 h conversion, and almost all of crude l-Glu were converted into GABA with a yield of 308.13 g L^−1^. Although the undetermined impurities were existed in crude l-Glu compared to chemical pure l-Glu, the conversion progressed smoothly with high GABA production and productivity (volumetric productivity: 25.68 g L^−1^ h^−1^, cell productivity: 24.75 g/g wet cell) (Fig. 6a). Notably, although our cell productivity was at the same level with the highest report (23–25 g/g wet cell), the volumetric productivity of our system was twice higher than that report (8–8.6 g L^−1^ h^−1^) (Plokhov et al. 2000). Moreover, that report used thermally activated cells (incubation at 53 °C for 1 h) and 0.04 mM PLP in the cluture medium, while we did not need any pretreatment and addition of expensive cofactor.

The reusability of biocatalyst is a key factor for the efficiency of the whole-cell bioconversion in industrial application. Due to the highest volumetric productivity from 2 M l-Glu as the substrate, cycling of the recombinant *E. coli* cells was investigated using 2 M crude l-Glu as the initial concentration (Fig. 6b). In cycle 1, all l-Glu was converted to GABA in 5 h with a yield of 204.87 g L^−1^. In cycle 2, the complete conversion only lasted 4 h with a yield of 205.13 g L^−1^. However, in cycle 3, the conversion time was increased to 6 h to achieve over 99 mol% conversion. Notably, cells disruption were observed during the bioconversion, and about 10 % or more cell loss after completion of each round (Additional file 1: Fig. S4). Thus, one batch of cells could be reused for at least three cycles at a conversion yield over 99 mol% using 2 M crude l-Glu, and the total GABA production reached 614.15 g L^−1^ within 15 h. The high production and productivity of our bioconversion is a promising cost-effective resource for GABA in industrial application.

## Conclusions

In this study, a process of GABA production from l-Glu using *E. coli* Δ*gabAB* overexpression of *L. lactis* GadB as whole-cell biocatalyst was developed. Without the addition of co-factor PLP, the highest GABA concentration was achieved at 308.96 g L^−1^ within 12 h, when engineered *E. coli* concentrated to an OD_600_ of 15 in 3 M l-Glu at 45 °C. Moreover, the engineered strain could be reused a three cycle successive conversion in 2 M crude Glu solution, and the total GABA yield reached 614.15 g L^−1^. This whole-cell biocatalytic system is a cost-effective process for industrial GABA production.

## Additional file

10.1186/s40064-016-2217-2 **Table S1.** Major chemical synthesis methods of GABA. Table S2. Plasmid copy number.** Fig. S1.** SDS-PAGE analysis of the soluble fraction cell extracts.** Fig. S2.** SDS-PAGE analysis of the supernatant from cell extracts.** Fig. S3.** l-Glu solubility in different concentrations of GABA solution.** Fig. S4.** Protein concentrations during each cycles of GABA production.
