# Supplementary material for: Whole-cell conversion of l-glutamic acid into gamma-aminobutyric acid by metabolically engineered Escherichia coli
Source: Springerplus. 2016 May 11;5:591. doi: 10.1186/s40064-016-2217-2 (PMC4864792; doi:10.1186/s40064-016-2217-2)
Supplement: Supplementary file 1 — 10.1186/s40064-016-2217-2 Table S1. Major chemical synthesis methods of GABA. Table S2. Plasmid copy number. Fig. S1. SDS-PAGE analysis of the soluble fraction cell extracts. Fig. S2. SDS-PAGE analysis of the supernatant from cell extracts. Fig. S3. l-Glu solubility in different concentrations of GABA solution. Fig. S4. Protein concentrations during each cycles of GABA production. [file 40064_2016_2217_MOESM1_ESM.docx]

**Additional files**

**Whole-cell conversion of** **glutamate into Gamma-aminobutyric**

**acid by metabolically engineered** ***Escherichia coli***

Chongrong Ke^a^, Xinwei Yang^a^, Huanxin Rao^a^, Wenchao Zeng^a^, Meirong Hu^b^, Yong Tao^b^, Jianzhong Huang^a*^

^a^National Engineering Research Center of Industrial Microbiology and Fermentation Technology ; College of Life Sciences, Fujian Normal University, Fuzhou, Fujian 350108, China

^b^CAS Key Laboratory of Microbial Physiological and Metabolic Engineering, Institute of Microbiology, Chinese Academy of Sciences, No. 1 West Beichen Road, Chaoyang District, Beijing 100101, China

**Tables**

**Table S1 Major chemical synthesis methods of GABA**

| **NO.** | **Reaction Process** | **Purification** |
| --- | --- | --- |
| **1** |  | **---** |
| **2** |  | **③①④②⑦** |
| **3** |  | **⑤①②⑥** |

Purification process: ①: distillation; ②: filtration; ③: centrifugation; ④: refrigeration; **⑤:** [decoloration](javascript:void(0);); ⑥: crystallization; ⑦: desiccation

**Table S2 Plasmid copy number used in this study**

| **NO.** | **Plasmid** | **Replicon** | **Copy number** |
| --- | --- | --- | --- |
| **1** | pYB1S | p15A | **18 ~ 22** |
| **2** | pRB1S | RSF1020 | **15 ~ 20** |
| **3** | pAB1S | colA | **20 ~ 40** |
| **4** | pDB1S | cloDF13 | **10 ~ 90** |
| **5** | pSB1S | pSC101 | **1 ~ 5** |
| **6** | pUB1S | colE1 | **300 ~ 500** |

**Figures**

**
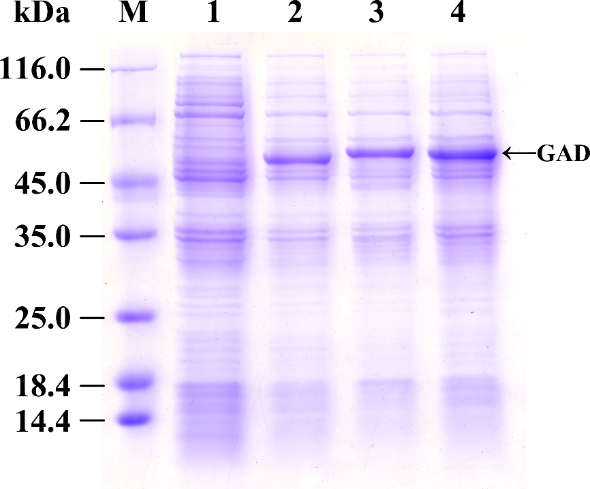
**

**Fig. S1** SDS-PAGE analysis of the soluble fraction cell extracts.

lane M, molecular weight markers; lane 1, *E. coli* BW25113 harboring pYB1s; lane 2, *E. coli* BW25113 harboring pYB-pgadB; lane 3, *E. coli* BW25113 harboring pYB-lgadB; lane 4, *E. coli* BW25113 harboring pYB-bgadB. The arrow indicates the GAD bands.


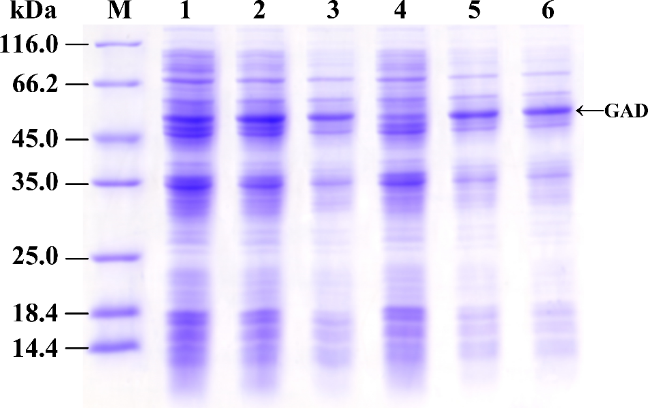

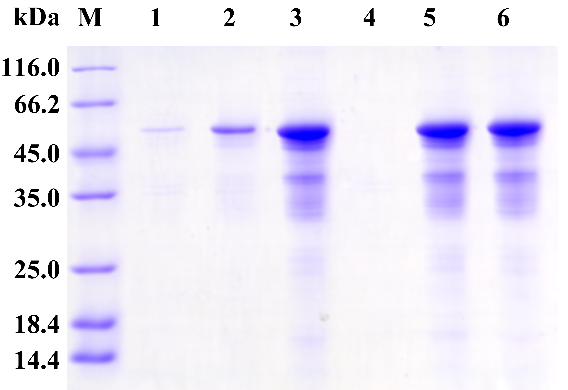


**Fig. S2** SDS-PAGE analysis of the supernatant from cell extracts

lane M, molecular weight markers; lane 1, *E. coli* harboring pRB-pgadB; lane 2, *E. coli* harboring pYB-pgadB; lane 3, *E. coli* harboring pAB-pgadB; lane 4, *E. coli* harboring pSB-pgadB; lane 5, *E. coli* harboring pDB-pgadB; lane 6, *E. coli* harboring pUB-pgadB; The arrow indicates the GAD bands.





**Fig. S3** L-Glu solubility in different concentrations of GABA solution





**Fig. S4** Protein concentrations during each cycles of GABA production
